# Evaluation of tumor response to cytokine-induced killer cells therapy in malignant solid tumors

**DOI:** 10.1186/s12967-014-0215-0

**Published:** 2014-08-12

**Authors:** Xiao-Dong Li, Mei Ji, Xiao Zheng, Zhong-Hua Ning, Jun Wu, Binfeng Lu, Chang-Ping Wu, Jing-Ting Jiang

**Affiliations:** Department of Oncology, The Third Affiliated Hospital of Soochow University, Changzhou, China; Department of Tumor Biological Treatment, The Third Affiliated Hospital of Soochow University, Changzhou, China; Department of Radiation Oncology, The Third Affiliated Hospital of Soochow University, Changzhou, China; Department of Immunology, University of Pittsburgh School of Medicine, Pittsburgh, USA

**Keywords:** CIK cells therapy, Tumor response, Evaluation system

## Abstract

CIK cells therapy has been evaluated as an adoptive cell immunotherapy for cancer patients, but there still have not been any standardized systems for evaluating the antitumor efficacy yet. The WHO and RECIST criteria have already been established for a few years but not sufficient to fully characterize the activity of immunotherapy. Based on these two criteria, the irRC was proposed for evaluating the efficacy of immunotherapy. A variety of bioassays for immune monitoring including the specific and non-specific methods, have been established. We recommend detect levels of various immunocytes, immune molecules and soluble molecules to find the correlations among them and clinicopathological characteristics to establish criteria for immunological classification. We also recommend a paradigm shift for the oncologists in the evaluation of immune therapies to ensure assessment of activity based on clinically relevant criteria and time points.

Malignancy has become a major cause of human deaths worldwide nowadays [1,2]. Unfortunately, traditional therapies including surgery, chemotherapy and radiotherapy often fail to eradicate tumor lesions completely and tend to result in many adverse events [3]. Thus, novel approaches for curing malignancies are urgently necessary.

In recent years, immunotherapy has emerged as an efficacious treatment modality with encouraging efficacy and slight adverse events in cancer therapy [4]. Among various kinds of immunotherapy, cytokine-induced killer (CIK) cells therapy has moved from the “bench to bedside” and been suggested as a promising method [5].

CIK cells, a subset of T lymphocytes with a natural killer T cell phenotype expressing both the CD3 and the CD56 markers, present potent non-major histocompatibility complex-restricted cytotoxicity against a variety of tumor target cells [6-15], which is similar to the NK cells [15]. The serial biological events following CIK cells administration to a cancer patient includes (a) immune activation and effective lymphocytes (mostly CD3^+^CD56^+^ T lymphocytes) proliferation starting early after the first administration, (b) clinically measurable antitumor effects mediated by activated immune cells over weeks to months, and (c) potential delayed effect on patient survival several months or even longer after the first administration [16].

CIK cells therapy has been evaluated as an adoptive cell immunotherapy for cancer patients in a number of clinical trials [13,17-26]. In our department, CIK cells therapy has been applied on more than 4000 cancer patients since late 90' of last century, and recently we have proposed a relatively standardized procedure CIK cells therapy [27]. And we have discussed the role of CIK cells infusion on immune enhancing [15]. But still, during the practice we found that there still have not been any standardized systems for evaluating the antitumor efficacy yet, while the promising efficacy of CIK cells on malignancies has been proved.

As widely acknowledged, the WHO and RECIST criteria have already been established for years in order to evaluate the efficacy of cytotoxic agents [28-31]. These criteria are not sufficient to fully characterize the activity of immunotherapy since most long-term responses are observed after an increase in tumor burden (TB) evaluated by WHO and RECIST criteria [32]. Nevertheless, CIK cells are not able to show abundant activity in a few weeks of the initial administration. More time is required for immunocytes expansion followed by infiltration of the tumor and a subsequent measurable antitumor effect [16], which means that radiographic evaluation of a progressive disease (PD) evaluated by the WHO or RECIST criteria does not necessarily mean therapeutic failure [33]. Furthermore, durable stable disease (SD) might also suggests that the appearance of measurable antitumor activity could take longer time for immune therapies than for cytotoxic therapies [34]. The facts reveal that the WHO or RECIST criteria are not suitable for evaluating the antitumor effect of CIK cells therapy.

Adjusting the clinical development paradigm from traditional therapies to immunotherapy requires a proper tool for evaluation, so the lack of standardized systems becomes an obvious shortcoming of employing CIK cells therapy. However, challenges are involved in each biological event mentioned above since the antitumor response induced by CIK cells therapy is not included in the traditional evaluation system [34]. so it is urgently needed to establish novel evaluation criteria for immune-related response. Another reason is that there can be huge discrepancies of the results of examinations for antitumor immunotherapy under various conditions or in different laboratories. For instance, it has been reported that a total of 36 laboratories used enzyme linked immunospot assay (ELISPOT) to examine the level of serum cytokine secreted by peripheral blood mononuclear cell of the same patient and the results vary from negative to strong positive [16]. The lack of a quality control measure for T cell–based assays that can be applied as a gold standard has hampered the establishment of correlation between the antitumor effect of immunotherapy including CIK cells therapy and clinical outcomes.

Considering of these and based on WHO and RECIST, a novel set of response criteria were evaluated in a few large multinational studies on advanced melanoma receiving ipilimumab, a fully human monoclonal antibody that blocks CTLA-4 [35,36]. In these studies, 4 distinct response patterns were observed: immediate response (response in baseline lesions-evident by week 12 with no new lesions), durable SD, response after TB increase (responses after an initial increase in total TB), and response in the presence of new lesions (a reduction in total TB during or after the appearance of new lesions later than week 12) [33]. Among these 4 response patterns, the first two are conventional, while the latter two are novel and specifically recognized by immunotherapeutic agents. Particularly, the results of one case study of the first novel tumor response pattern reveal that TB firstly increases and then decreases to a complete response. Importantly, all these 4 patterns seem to be associated with promising survival outcome compared with patients with PD evaluated by WHO criteria [33]. In fact, it has been found that PD (the appearance of new lesions or increase in the size of existing lesions) might be resulted by just lymphocytic infiltration but not the represent of true disease progression [37,38].

In order to create a process which systematically captures all observed response patterns, the irRC, generally based on the WHO and RECIST criteria but do not require a substantial departure from standard oncology practice, was proposed by Cancer Immunotherapy Consortium of the Cancer Research Institute [33,39]. The irRC develops a novelty in the measurement of new lesions that are included in the total TB [40]. So generally, the irRC provides a means of accounting for delayed changes in TB through confirmation of progression at subsequent time points.

The overall response according to the irRC is derived from time-point response assessments (based on total TB) as irCR, complete disappearance of all lesions confirmation by a repeat, consecutive assessment no less than 4 weeks from the date first recorded; irPR, decrease in TB ≥ 50% relative to baseline confirmed by a consecutive assessment at least 4 weeks after first record; irSD, not matching the criteria for irCR or irPR, in absence of irPD; irPD, increase in TB ≥ 25% relative to minimum recorded total TB confirmation by a repeat, consecutive assessment no less than 4 weeks from the date first recorded [33].

The irRC were defined based on data from ipilimumab clinical trials, but their conceptual foundations result from consistent observations with several agents across the immune therapy community, and therefore it is expected that these criteria will have broad applicability to other immunotherapeutic agents. However, it is inappropriate to copy and apply the irRC mechanically for the pattern of tumor response to CIK cells therapy in malignant solid tumors, adjustment is required.

A variety of bioassays for immune monitoring, including the specific and non-specific methods, has been established. The specific and non-specific methods include ELISPOT assay and cytometry-based tests such as intracellular cytokine staining, HLA-peptide multimer staining, and the carboxyfluorescein succinimidyl ester assay [41-44], and delayed type hypersensitivity method is applied to detect the existence of antibody-specific T lymphocytes *in vivo* after treatment [45], and soluble-MHC-petide tetramer methods are used for the amount of T lymphocytes [42]. Non-specific immune responses include flow cytometry determining peripheral blood lymphocytes subtypes, and enzyme linked immunosorbent assay or cytometric bead array methods determining the serum level of some cytokines. Even though the fundamentals of these assays have been well established, a plethora of different laboratory protocols is used, which leads to that results vary in a very wide spectrum [44]. However fortunately, the change of T lymphocytes subtypes after CIK cells administration in various cancers were similar in different laboratories.

Though antitumor immunotherapy achieves the antitumor effect by activating immune responses, it is still not clear whether the clinical outcome is directly related with the immune responses. Nijman *et al.* used ELISPOT method to detect p53-specific T lymphocytes, and no relevance between the therapeutic efficacy and T lymphocyte-induced response was observed in this phase II clinical trial on ovarian cancer [46]. But Weiner *et al.* applied the combination regimen of peptide vaccine and GM-CSF+/-IFN-α2b in the treatment of 120 cases of advanced malignant melanoma enrolled in a stage II clinical trial with an average follow-up of 25.4 months, and found that those who had a specific immune response enjoyed a significantly longer overall survival time than those did not (21.3 *vs.* 13.4 months, *P* = 0.046), suggesting that the overall survival time after the immunotherapy was related to the immune response [47]. Additionally, the inability to use cellular immune response assays to define biomarkers and to investigate their correlation with clinical outcomes has its roots in highly variable and often nonreproducible assay results in multicenter trials [48,49]. Thus, adequate indicators which reflect immune response could possibly be biomarkers for evaluating the efficacy of CIK cells therapy. But the challenge is to determine which biomarkers have the greatest potential to be investigated as correlates to clinical response. The ideal immunologic biomarkers should be one which can (a) be measured easily from bodily fluids, (b) is quantitative allowing for stratification of patients based on magnitude of response and allowed some qualitative assessment of the response, and (c) reflect the mechanism of action of the agent studied or the direct effect of cancer immunity [50].

There are indicators in the published literature that blood-based immunologic biomarkers that predict clinical response can be developed [50]. We recommend detect levels of various immunocytes, immune molecules and soluble molecules, and find the correlations between them and clinicopathological characteristics to establish standards for immunological classification. That is why we perform CIK cells therapy and analyze the correlation between the therapeutic efficacy and these levels to screen out the immunocytes and proportions of immunocyte subsets which directly affect the therapeutic efficacies.

Upon the clinical practice, we therefore recommend a paradigm shift for the oncologist in the evaluation of immune therapies to ensure assessment of activity based on clinically relevant criteria and time points.

1. *The overall survival and progression-free survival time*: the main parameters for evaluation
2. *Regular assessment*
  1. CIK cells therapy response: evaluated by irRC
  2. Tumor markers (vary for different kinds of malignancies)
  3. Patients’ status of quality of life (referring to RECIST and WHO criteria)
3. *Immune response monitoring*
  1. Percentages and absolute values of T lymphocytes including CD3^+^, CD4^+^, CD8^+^CD3^+^CD4^+^ and CD3^+^CD8^+^ lymphocytes, Th1/Th17/Th2/Treg cells, dendritic cells,
  2. Percentages and absolute values of CD3^+^ CD56^+^ lymphocytes (the )
  3. Toxicity of NK cells
  4. Granzymes and perforins of CD8^+^ T cells and γδT cells
  5. Phenotypes of CD3^+^CD8^+^ T cells, CD3^+^CD4^+^ T cells and γδT cells
  6. Detection of CD3^+^CD4^+^Foxp3^+^ T cells
  7. Activation marker including CD3^+^HLA^-^ DR, CD4^+^HLA^-^ DR and CD8^+^HLA^-^ DR
  8. Detection of antigen-specific T cells (CD4^+^ and CD8^+^ T cells)
  9. CD4^+^ CD25^+^ Treg cells [51-53]
4. *Soluble molecules levels monitoring*
  1. Negative regulating cytokines: IL-4, IL-10, TGF-β and VEGF.
  2. Positive regulating cytokines: IL-2, IL-12, IFN-γ and TNF-α.
  3. T lymphocytes-regulating molecules including B7-H4 [54], B7-H3, PD-L1 [55]

The indicators should be recruited anytime when adequate novel ones are found, and need to be verified in clinical trials enrolling large sample sizes.

Besides, the presence of CIK-related adverse events has been shown to be predictive of better clinical responses and outcomes. On our experience, most patients receiving CIK cells therapy had an improvement in their appetite, physical strength, sleeping, pain remission. There were rarely severe adverse events noted. All moderate adverse events disappeared after allopathic treatments. So the occurrence of adverse events cannot be enrolled to measure the tumor response to CIK cells therapy.

The final but the most point is that these items mentioned above can be scored separately, and added together then a scoring system is formed. The most important point is to 1) to avoid the impact of prior treatments on CIK cells therapy and 2) find the adequate weight of each and every item.

## Future perspective

Conclusively, the evaluation of therapeutic effect of CIK cells therapy is based on the irRC but not restricted to it. We recommend detect broad sorts of indexes and use various methods to improve the evaluation. But our recommendation is still far from perfection. Novel, adequate methods should be verified in practice and more indicators might be recruited. And a scoring system is required but the score of each item should be verified. Progressive clinical trials with large sample sizes should be performed and provide the evidences for applying the criterion.

## References

[1] Siegel R, DeSantis C, Virgo K, Stein K, Mariotto A, Smith T, Cooper D, Gansler T, Lerro C, Fedewa S, Lin C, Leach C, Cannady RS, Cho H, Scoppa S, Hachey M, Kirch R, Jemal A, Ward E (2012). Cancer treatment and survivorship statistics, 2012. CA Cancer J Clin.

[2] Siegel R, Naishadham D, Jemal A (2012). Cancer statistics, 2012. CA Cancer J Clin.

[3] Hontscha C, Borck Y, Zhou H, Messmer D, Schmidt-Wolf IG (2011). Clinical trials on CIK cells: first report of the international registry on CIK cells (IRCC). J Cancer Res Clin Oncol.

[4] Stroncek D, Berlyne D, Fox B, Gee A, Heimfeld S, Lindblad R, Loper K, McKenna D, Rooney C, Sabatino M, Wagner E, Whiteside T, Wood D, Heath-Mondoro T (2010). Developments in clinical cell therapy. Cytotherapy.

[5] Linn YC, Hui KM (2010). Cytokine-induced NK-like T cells: from bench to bedside. J Biomed Biotechnol.

[6] Bradley M, Zeytun A, Rafi-Janajreh A, Nagarkatti PS, Nagarkatti M (1998). Role of spontaneous and interleukin-2-induced natural killer cell activity in the cytotoxicity and rejection of Fas + and Fas- tumor cells. Blood.

[7] Heusel JW, Wesselschmidt RL, Shresta S, Russell JH, Ley TJ (1994). Cytotoxic lymphocytes require granzyme B for the rapid induction of DNA fragmentation and apoptosis in allogeneic target cells. Cell.

[8] Joshi PS, Liu JQ, Wang Y, Chang X, Richards J, Assarsson E, Shi FD, Ljunggren HG, Bai XF (2006). Cytokine-induced killer T cells kill immature dendritic cells by TCR-independent and perforin-dependent mechanisms. J Leukoc Biol.

[9] Kagi D, Ledermann B, Burki K, Seiler P, Odermatt B, Olsen KJ, Podack ER, Zinkernagel RM, Hengartner H (1994). Cytotoxicity mediated by T cells and natural killer cells is greatly impaired in perforin-deficient mice. Nature.

[10] Linn YC, Lau LC, Hui KM (2002). Generation of cytokine-induced killer cells from leukaemic samples with in vitro cytotoxicity against autologous and allogeneic leukaemic blasts. Br J Haematol.

[11] Schmidt-Wolf IG, Lefterova P, Johnston V, Scheffold C, Csipai M, Mehta BA, Tsuruo T, Huhn D, Negrin RS (1996). Sensitivity of multidrug-resistant tumor cell lines to immunologic effector cells. Cell Immunol.

[12] Schmidt-Wolf IG, Negrin RS, Kiem HP, Blume KG, Weissman IL (1991). Use of a SCID mouse/human lymphoma model to evaluate cytokine-induced killer cells with potent antitumor cell activity. J Exp Med.

[13] Wang FS, Liu MX, Zhang B, Shi M, Lei ZY, Sun WB, Du QY, Chen JM (2002). Antitumor activities of human autologous cytokine-induced killer (CIK) cells against hepatocellular carcinoma cells in vitro and in vivo. World J Gastroenterol.

[14] Zhang YS, Yuan FJ, Jia GF, Zhang JF, Hu LY, Huang L, Wang J, Dai ZQ (2005). CIK cells from patients with HCC possess strong cytotoxicity to multidrug-resistant cell line Bel-7402/R. World J Gastroenterol.

[15] Jiang J, Wu C, Lu B (2013). Cytokine-induced killer cells promote antitumor immunity. J Transl Med.

[16] Hoos A, Eggermont AM, Janetzki S, Hodi FS, Ibrahim R, Anderson A, Humphrey R, Blumenstein B, Old L, Wolchok J (2010). Improved endpoints for cancer immunotherapy trials. J Natl Cancer Inst.

[17] Kuci S, Rettinger E, Voss B, Weber G, Stais M, Kreyenberg H, Willasch A, Kuci Z, Koscielniak E, Kloss S, von Laer D, Klingebiel T, Bader P (2010). Efficient lysis of rhabdomyosarcoma cells by cytokine-induced killer cells: implications for adoptive immunotherapy after allogeneic stem cell transplantation. Haematologica.

[18] Linn YC, Hui KM (2003). Cytokine-induced killer cells: NK-like T cells with cytotolytic specificity against leukemia. Leuk Lymphoma.

[19] Shi M, Zhang B, Tang ZR, Lei ZY, Wang HF, Feng YY, Fan ZP, Xu DP, Wang FS (2004). Autologous cytokine-induced killer cell therapy in clinical trial phase I is safe in patients with primary hepatocellular carcinoma. World J Gastroenterol.

[20] Koscielniak E, Gross-Wieltsch U, Treuner J, Winkler P, Klingebiel T, Lang P, Bader P, Niethammer D, Handgretinger R (2005). Graft-versus-Ewing sarcoma effect and long-term remission induced by haploidentical stem-cell transplantation in a patient with relapse of metastatic disease. J Clin Oncol.

[21] Leemhuis T, Wells S, Scheffold C, Edinger M, Negrin RS (2005). A phase I trial of autologous cytokine-induced killer cells for the treatment of relapsed Hodgkin disease and non-Hodgkin lymphoma. Biol Blood Marrow Transplant.

[22] Linn YC, Wang SM, Hui KM (2005). Comparative gene expression profiling of cytokine-induced killer cells in response to acute myloid leukemic and acute lymphoblastic leukemic stimulators using oligonucleotide arrays. Exp Hematol.

[23] Chan JK, Hamilton CA, Cheung MK, Karimi M, Baker J, Gall JM, Schulz S, Thorne SH, Teng NN, Contag CH, Lum LG, Negrin RS (2006). Enhanced killing of primary ovarian cancer by retargeting autologous cytokine-induced killer cells with bispecific antibodies: a preclinical study. Clin Cancer Res.

[24] Jiang J, Xu N, Wu C, Deng H, Lu M, Li M, Xu B, Wu J, Wang R, Xu J, Nilsson-Ehle P (2006). Treatment of advanced gastric cancer by chemotherapy combined with autologous cytokine-induced killer cells. Anticancer Res.

[25] Jiang JT, Wu CP, Shi LR, Xu N, Deng HF, Lu MY, Ji M, Zhu YB, Zhang XG (2008). Side Effects during Treatment of Advanced Gastric Carcinoma by Chemotherapy Combined with CIK-cell Transfusion in Elderly People. Clin Oncol Cancer Res.

[26] Wu C, Jiang J, Shi L, Xu N (2008). Prospective study of chemotherapy in combination with cytokine-induced killer cells in patients suffering from advanced non-small cell lung cancer. Anticancer Res.

[27] Li XD, Xu B, Wu J, Ji M, Xu BH, Jiang JT, Wu CP (2012). Review of Chinese clinical trials on CIK cell treatment for malignancies. Clin Transl Oncol.

[28] World Health Organization (1979). WHO handbook for reporting results of cancer treatment.

[29] Van Hoe L, Van Cutsem E, Vergote I, Marchal G, Baert AL (1996). Reporting on the results of cancer treatment in patients with metastatic liver disease: proposal of symmetric size-dependent CT-criteria for response. Ann Oncol.

[30] Therasse P, Arbuck SG, Eisenhauer EA, Wanders J, Kaplan RS, Rubinstein L, Verweij J, Van Glabbeke M, van Oosterom AT, Christian MC, Gwyther SG (2000). New guidelines to evaluate the response to treatment in solid tumors. European Organization for Research and Treatment of Cancer, National Cancer Institute of the United States, National Cancer Institute of Canada. J Natl Cancer Inst.

[31] Eisenhauer EA, Therasse P, Bogaerts J, Schwartz LH, Sargent D, Ford R, Dancey J, Arbuck S, Gwyther S, Mooney M, Rubinstein L, Shankar L, Dodd L, Kaplan R, Lacombe D, Verweij J (2009). New response evaluation criteria in solid tumours: revised RECIST guideline (version 1.1). Eur J Cancer.

[32] Melero I, Hervas-Stubbs S, Glennie M, Pardoll DM, Chen L (2007). Immunostimulatory monoclonal antibodies for cancer therapy. Nat Rev Cancer.

[33] Wolchok JD, Hoos A, O'Day S, Weber JS, Hamid O, Lebbe C, Maio M, Binder M, Bohnsack O, Nichol G, Humphrey R, Hodi FS (2009). Guidelines for the evaluation of immune therapy activity in solid tumors: immune-related response criteria. Clin Cancer Res.

[34] Hoos A, Parmiani G, Hege K, Sznol M, Loibner H, Eggermont A, Urba W, Blumenstein B, Sacks N, Keilholz U, Nichol G (2007). A clinical development paradigm for cancer vaccines and related biologics. J Immunother.

[35] Wolchok JD, Neyns B, Linette G, Negrier S, Lutzky J, Thomas L, Waterfield W, Schadendorf D, Smylie M, Guthrie T, Grob JJ, Chesney J, Chin K, Chen K, Hoos A, O'Day SJ, Lebbé C (2010). Ipilimumab monotherapy in patients with pretreated advanced melanoma: a randomised, double-blind, multicentre, phase 2, dose-ranging study. Lancet Oncol.

[36] Weber J, Thompson JA, Hamid O, Minor D, Amin A, Ron I, Ridolfi R, Assi H, Maraveyas A, Berman D, Siegel J, O'Day SJ (2009). A randomized, double-blind, placebo-controlled, phase II study comparing the tolerability and efficacy of ipilimumab administered with or without prophylactic budesonide in patients with unresectable stage III or IV melanoma. Clin Cancer Res.

[37] Hodi FS, Butler M, Oble DA, Seiden MV, Haluska FG, Kruse A, Macrae S, Nelson M, Canning C, Lowy I, Korman A, Lautz D, Russell S, Jaklitsch MT, Ramaiya N, Chen TC, Neuberg D, Allison JP, Mihm MC, Dranoff G (2008). Immunologic and clinical effects of antibody blockade of cytotoxic T lymphocyte-associated antigen 4 in previously vaccinated cancer patients. Proc Natl Acad Sci U S A.

[38] Hodi FS, Oble DA, Drappatz J, Velazquez EF, Ramaiya N, Ramakrishna N, Day AL, Kruse A, Mac Rae S, Hoos A, Mihm M (2008). CTLA-4 blockade with ipilimumab induces significant clinical benefit in a female with melanoma metastases to the CNS. Nat Clin Pract Oncol.

[39] Tuma RS (2008). New response criteria proposed for immunotherapies. J Natl Cancer Inst.

[40] Hodi FS, Hoos A, Ibrahim R, Chin K, Pehamberger H, Harmankaya K, O'Day S, Hamid O, Humphrey R, Wolchok J (2008). Novel efficacy criteria for antitumor ac-tivity to immunotherapy using the example of ipilimumab, and anti-CTLA-4 monoclonal antibody [abstract 3008]. J Clin Oncol.

[41] Berd D, Sato T, Cohn H, Maguire HC, Mastrangelo MJ (2001). Treatment of metastatic melanoma with autologous, hapten-modified melanoma vaccine: regression of pulmonary metastases. Int J Cancer.

[42] Hobeika AC, Morse MA, Osada T, Ghanayem M, Niedzwiecki D, Barrier R, Lyerly HK, Clay TM (2005). Enumerating antigen-specific T-cell responses in peripheral blood: a comparison of peptide MHC Tetramer, ELISpot, and intracellular cytokine analysis. J Immunother.

[43] Walker EB, Disis ML (2003). Monitoring immune responses in cancer patients receiving tumor vaccines. Int Rev Immunol.

[44] Keilholz U, Weber J, Finke JH, Gabrilovich DI, Kast WM, Disis ML, Kirkwood JM, Scheibenbogen C, Schlom J, Maino VC, Lyerly HK, Lee PP, Storkus W, Marincola F, Worobec A, Atkins MB (2002). Immunologic monitoring of cancer vaccine therapy: results of a workshop sponsored by the Society for Biological Therapy. J Immunother.

[45] Sato Y, Shomura H, Maeda Y, Mine T, Une Y, Akasaka Y, Kondo M, Takahashi S, Shinohara T, Katagiri K, Sato M, Okada S, Matsui K, Yamada A, Yamana H, Itoh K, Todo S (2003). Immunological evaluation of peptide vaccination for patients with gastric cancer based on pre-existing cellular response to peptide. Cancer Sci.

[46] Leffers N, Lambeck AJ, Gooden MJ, Hoogeboom BN, Wolf R, Hamming IE, Hepkema BG, Willemse PH, Molmans BH, Hollema H, Drijfhout JW, Sluiter WJ, Valentijn AR, Fathers LM, Oostendorp J, van der Zee AG, Melief CJ, van der Burg SH, Daemen T, Nijman HW (2009). Immunization with a P53 synthetic long peptide vaccine induces P53-specific immune responses in ovarian cancer patients, a phase II trial. Int J Cancer.

[47] Kirkwood JM, Lee S, Moschos SJ, Albertini MR, Michalak JC, Sander C, Whiteside T, Butterfield LH, Weiner L (2009). Immunogenicity and antitumor effects of vaccination with peptide vaccine+/-granulocyte-monocyte colony-stimulating factor and/or IFN-alpha2b in advanced metastatic melanoma: Eastern Cooperative Oncology Group Phase II Trial E1696. Clin Cancer Res.

[48] Janetzki S, Britten CM, Kalos M, Levitsky HI, Maecker HT, Melief CJ, Old LJ, Romero P, Hoos A, Davis MM (2009). "MIATA"-minimal information about T cell assays. Immunity.

[49] Britten CM, Janetzki S, van der Burg SH, Gouttefangeas C, Hoos A (2008). Toward the harmonization of immune monitoring in clinical trials: quo vadis?. Cancer Immunol Immunother.

[50] Disis ML (2011). Immunologic biomarkers as correlates of clinical response to cancer immunotherapy. Cancer Immunol Immunother.

[51] Li H, Yu JP, Cao S, Wei F, Zhang P, An XM, Huang ZT, Ren XB (2007). CD4 + CD25 + regulatory T cells decreased the antitumor activity of cytokine-induced killer (CIK) cells of lung cancer patients. J Clin Immunol.

[52] Niam M, Linn YC, Fook Chong S, Lim TJ, Chu S, Choong A, Yong HX, Suck G, Chan M, Koh M (2011). Clinical scale expansion of cytokine-induced killer cells is feasible from healthy donors and patients with acute and chronic myeloid leukemia at various stages of therapy. Exp Hematol.

[53] Pan CC, Huang ZL, Li W, Zhao M, Zhou QM, Xia JC, Wu PH (2010). Serum alpha-fetoprotein measurement in predicting clinical outcome related to autologous cytokine-induced killer cells in patients with hepatocellular carcinoma undergone minimally invasive therapy. Chin J Cancer.

[54] Jiang J, Zhu Y, Wu C, Shen Y, Wei W, Chen L, Zheng X, Sun J, Lu B, Zhang X (2010). Tumor expression of B7-H4 predicts poor survival of patients suffering from gastric cancer. Cancer Immunol Immunother.

[55] Wu C, Zhu Y, Jiang J, Zhao J, Zhang XG, Xu N (2006). Immunohistochemical localization of programmed death-1 ligand-1 (PD-L1) in gastric carcinoma and its clinical significance. Acta Histochem.

